# Large‐scale reconfiguration of connectivity patterns among attentional networks during context‐dependent adjustment of cognitive control

**DOI:** 10.1002/hbm.25467

**Published:** 2021-05-14

**Authors:** Yilu Li, Yanqing Wang, Fangwen Yu, Antao Chen

**Affiliations:** ^1^ Key Laboratory of Cognition and Personality of Ministry of Education, Faculty of Psychology Southwest University Chongqing China; ^2^ Institute of Psychology Chinese Academy of Sciences and University of Chinese Academy of Sciences Beijing China; ^3^ Key Laboratory for Neuroinformation, Center for Information in Medicine University of Electronic Science and Technology of China Chengdu China

**Keywords:** attentional networks, cognitive control, connectivity patterns, reconfiguration

## Abstract

The ability to adjust our behavior flexibly depending on situational demands and changes in the environment is an important characteristic of cognitive control. Previous studies have proved that this type of adaptive control plays a crucial role in selective attention, but have barely explored whether and how attentional networks support adaptive control. In the present study, a Stroop task with a different proportion of incongruent trials was used to investigate the brain activity and connectivity of six typical attentional control networks (i.e., the fronto‐parietal network (FPN), cingulo‐opercular network (CON), default mode network (DMN), dorsal attention network (DAN), and ventral attention network/salience network (VAN/SN)) in the environment with changing control demand. The behavioral analysis indicated a decreased Stroop interference (incongruent vs. congruent trial response time [RT]) with the increase in the proportion of incongruent trials within a block, indicating that cognitive control was improved there. The fMRI data revealed that the attenuate Stroop interference was accompanied by the activation of frontal and parietal regions, such as bilateral dorsolateral prefrontal cortex and anterior cingulate cortex. Crucially, the improved cognitive control induced by the increased proportion of incongruent trials was associated with the enhanced functional connectivity within the five networks, and a greater connection between CON with the DAN/SN, and between DMN with the CON/DAN/SN. Meanwhile, however, the functional coupling between the FPN and VAN was decreased. These results suggest that flexible regulations of cognitive control are implemented by the large‐scale reconfiguration of connectivity patterns among the attentional networks.

## Graphical abstract

Flexible regulations of cognitive control supported by functional connectivity changes among the attentional networks. Coupling within and between cingulo‐opercular network and dorsal attention network underpin the proactive control implemented in contexts that require control frequently. Cognitive control reactively implemented in contexts that require control rarely associated with functional coupling between the fronto‐parietal network and ventral attention network.

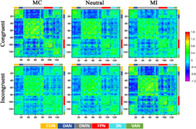

## 1 INTRODUCTION

Cognitive control supports flexible goal‐directed behavior by dynamically allocating attention resources to enhance task‐relevant information according to current goals and intentions (Cai et al., 2016; Miller & Cohen, 2001). Proactive adjustment of conflict‐control processes is a prominent function of cognitive control that serves to adapt strategically to changing control demands, to reduce the need to adjust cognitive control reactively in forthcoming trials (Jiang, Beck, Heller, & Egner, 2015; Jiang, Heller, & Egner, 2014; Muhle‐Karbe, Jiang, & Egner, 2018).

In particular, previous studies have examined many task designs with the aim of measuring the flexible adaptable control to various contextual factors, including temporal context (Aben et al., 2019), category (Bugg & Dey, 2018), and individual items (Blais & Bunge, 2010; Chiu, Jiang, & Egner, 2017; Jiang et al., 2015). For instance, using a Stroop paradigm, a proportion congruency (PC) effect was observed that was indexed by a reduction in Stroop interference (incongruent vs. congruent trial response time [RT]) in blocks with mostly incongruent (MI) trials (e.g., 75% incongruent trials and 25% congruent trials) relative to blocks with mostly congruent (MC) trials (e.g., 25% incongruent trials and 75% congruent trials) (Blais & Bunge, 2010; Braem et al., 2019; Bugg, Jacoby, & Toth, 2008; Jiang et al., 2014; Jiang et al., 2015). This suggests that humans can learn and apply the statistical regularities of cognitive control demand in the temporal context (e.g., blocks of trials) to predict the forthcoming demand of cognitive control and adjust cognitive processes accordingly.

Over the past few years, our understanding of the various neural processes underlying the manner in which the human attention system self‐adjusts to optimize performance has advanced. For instance, previous research has demonstrated that multiple key areas in the attention system, such as the lateral prefrontal cortex (lPFC), dorsal anterior cingulate cortex (ACC), insula, and parietal cortex, are involved in adjustment to a varying control demand according to learned rules and contextual information (Aben et al., 2019; Blais & Bunge, 2010; Chiu & Egner, 2019; De Pisapia & Braver, 2006; Jiang et al., 2015). Notably, recent studies have proposed that the neural activities recorded while performing a task are not only reflected by a change in neural activity within brain areas, but also by changes in functional interactions among large‐scale brain networks (Bassett et al., 2011; Bassett, Yang, Wymbs, & Grafton, 2015; Cole, Bassett, Power, Braver, & Petersen, 2014; Mohr et al., 2016).

Attentional control often recruits the fronto‐parietal network (FPN), the cingulo‐opercular network (CON), the default mode network (DMN), the attention network including the dorsal attention network (DAN), and the ventral attention network/salience network (VAN/SN) (Cocchi, Zalesky, Fornito, & Mattingley, 2013; Dosenbach, Fair, Cohen, Schlaggar, & Petersen, 2008; Power et al., 2011). A growing body of evidence has shown that these six networks may form two separate control mechanisms, which allows sustained and flexible attentional control at phasic and tonic timescales (Dosenbach et al., 2008; Palenciano, González‐García, Arco, & Ruz, 2019). In terms of cognitive control processes, the CON provides preparatory and sustained activity and is involved in proactive control (Dosenbach et al., 2006; Dosenbach et al., 2007; Dosenbach et al., 2008; Palenciano et al., 2019), whereas the FPN is associated with reactive processing adjustment and participates mainly in a transient and flexible manner (Dosenbach et al., 2006; Dosenbach et al., 2007; Dosenbach et al., 2008; Palenciano et al., 2019). Conversely, the DMN is considered as the task‐negative network as it contributes to the introspective process but is inversely correlated with cognitive control (Dixon et al., 2018; Lawrence, Ross, Hoffmann, Garavan, & Stein, 2003). Moreover, the DAN modulates attentional processing in preparation for expected input via top‐down processing, the VAN is responsible for detecting unexpected events in the changing environment, and the SN supports detecting behaviorally relevant stimuli and maintains a priority map of the visual environment (Corbetta, Patel, & Shulman, 2008; Dajani & Uddin, 2015; Farrant & Uddin, 2015; Kim, 2014). These attentional networks have been shown to play a meaningful role in other cognitive tasks, such as reasoning (Hearne, Cocchi, Zalesky, & Mattingley, 2017) and permuted role operations (Cole et al., 2013). However, whether and how the attention networks and control networks mentioned above cooperate to support varying control demands remains unclear.

By manipulating the statistical regularities of conflicts, previous studies revealed that the proactive and reactive control processes, which operated depending on temporal context, shared the same brain areas (Aben et al., 2019); or explored the key subcortical structure for control learning among different cognitive control demands that were bundled with the proportion of incongruent trials in the block (Jiang et al., 2015). By referring to these designs, we manipulated the contextual control demand by varying the block‐wise relative frequencies of incongruent trials and congruent trials in a classic word‐color Stroop task that has been used widely in cognitive control research (Braem et al., 2019; Kane & Engle, 2003; Torres‐Quesada, Lupianez, Milliken, & Funes, 2014). In addition, a psychophysiological interaction (PPI) analysis (Di & Biswal, 2019) was performed to examine the changes in brain network connectivity induced by the control demand among these attentional networks. Ultimately, we examined the links between the behavioral PC effect and connectivity patterns within and between attentional networks (CON, FPN, DMN, DAN, and VAN/SN). We hypothesized that the reconfiguration of connectivity patterns of attentional networks supports flexible adjustment of cognitive control in response to varying control demands. In particular, we predicted that, as the proportion of incongruent trials increased, proactive control would dominate and be supported by stronger connections between the CON and DAN so as to form sustained anticipation and prevention of conflict with enhanced top‐down processes; nevertheless, in cases where conflict rarely occurs, reactive control could be increasingly involved in detecting and resolving conflict in a more economical way, which might be based on enhanced connectivity between the FPN and VAN.

## 2 MATERIALS AND METHODS

### 2.1 Participants

Thirty‐two healthy right‐handed participants with normal or corrected‐to‐normal visual acuity and color vision took part in the experiment. All participants reported no history of psychiatric or neurological disorders. Two of them were excluded due to excessive head movement (>2 mm), leaving 30 participants (14 females; age, 18–26; mean ± *SD*, 21.54 ± 2.19 years). Before participation, all participants provided written informed consent that was approved by the ethics committee of Southwest University, China.

### 2.2 Stimuli and design

**FIGURE 1:**
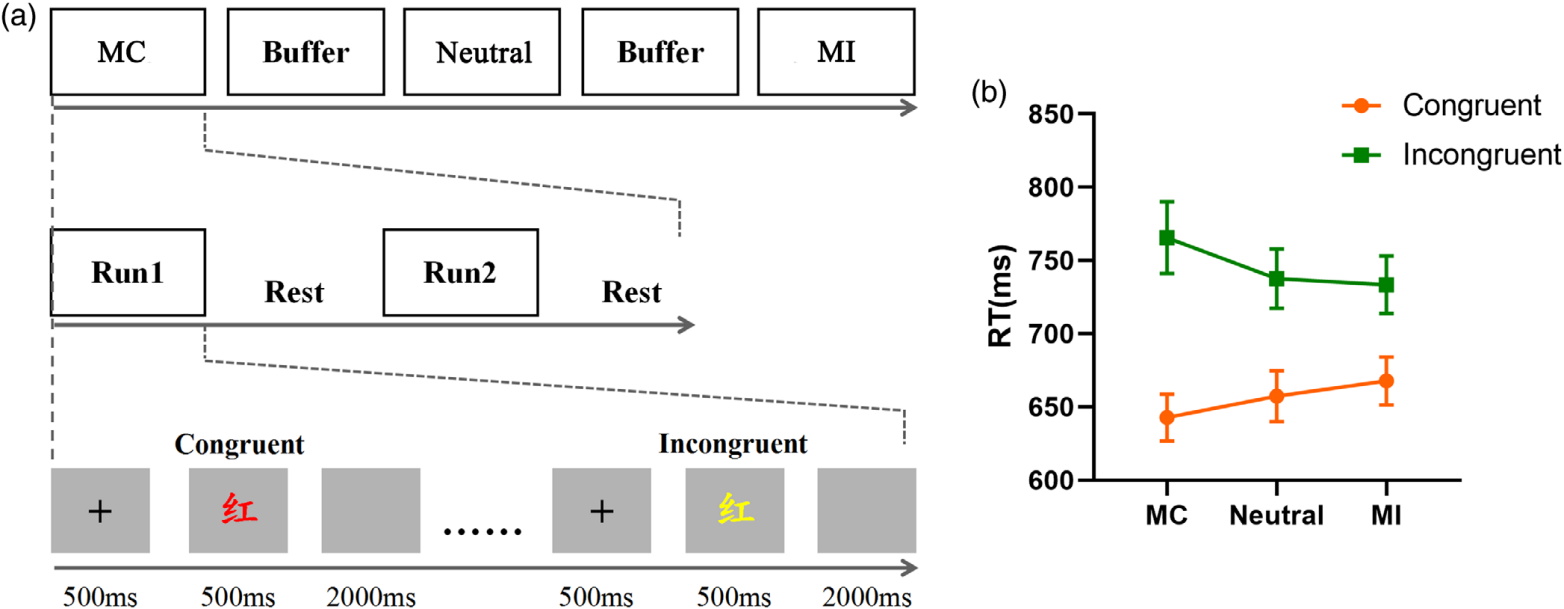
The experimental design (a) and behavioral results (b). Congruent refers to congruent trials, and incongruent refers to incongruent trials. The Chinese character “红” means red. RT, reaction time; MC, mostly congruent blocks (i.e., 75% congruent trials and 25% incongruent trials); neutral, blocks containing equal number of congruent and incongruent trials; MI, mostly incongruent blocks (i.e., 25% congruent trials and 75% incongruent trials)

Participants performed a Stroop task in which the ink color of color words was categorized as fast as possible by pressing the corresponding button with their middle and index fingers of the left and right hands, while trying to ignore the task‐irrelevant semantic meaning. Task stimuli consisted of four Chinese words (i.e., “Lan” [blue, RGB: 0, 0, 255], “Huang” [yellow, RGB: 255, 255, 0], “Hong” [red, RGB: 255, 0, 0), and “Lv” [green, RGB: 0, 255, 0]) that were presented in the semantically corresponding font color (congruent trials) or in a different font color (incongruent trials) in the center of a gray background (visual angle, 5° × 6°). During each trial, the participant stared at the presentation of a fixation cross in the gray background color for 500 ms, which was followed by the presentation of a word target stimulus shown for 500 ms and then replaced by a gray screen for 2000 ms (Figure 1(a)). Responses were recorded for a duration of 1,500 ms after target onset, with an intertrial interval of 1,000 ms.

Participants carried out three types of blocks with a varying proportion of incongruent trials: blocks containing 25% incongruent trials (MC blocks), with the lowest likelihood of experiencing conflict; blocks comprising 50% congruent trials and 50% incongruent trials (neutral blocks, neutral); and blocks containing 75% incongruent trials (MI blocks), with the highest probability of experiencing conflict. No participant was informed about the block type manipulation in the task. Each block type was considered as a unique context with a specific intensity of cognitive control demand that was augmented as the proportion of incongruent trials increased (Jiang et al., 2015; Jiang et al., 2020). Two runs per context (Figure 1(a)), with each run including an equal number of trials (120 trials), in another word, each context consists of equally 240 trials. The order of the three different contexts (i.e., three block types) was counterbalanced among participants, and the order of the trials was randomized in run level for each participant. As the PC effect has a sustained nature, the formed effect would be transferred to the subsequent block (Torres‐Quesada, Funes, & Lupiáñez, 2013). To control this carry‐over effect between MC and MI blocks, a buffer block consisting of 50% incongruent trials over 80 trials was included between different contexts. Moreover, a practice test with 80 trials in 50% congruency was conducted for all participants before the task and ended when 80% accuracy was achieved.

### 2.3 fMRI image acquisition

Functional MRI scanning was acquired with a 3.0‐T Siemens scanner (Siemens Magnetom Trio TIM, Erlangen, Germany). Functional scans consisted of 245 volumes per run recorded using a T2‐weighted echo planar imaging sequence of 24 axial slices (repetition time [TR] = 1,500 ms; echo time [TE] = 30 ms; flip angle = 90°; resolution matrix = 64 × 64; field of view = 192 × 192 mm^2^; voxel size = 3 × 3 × 5 mm^3^). Anatomical images consisted of 176 slices acquired with a thickness of 1 mm (TR = 1,900 ms; TE = 2.52 ms; flip angle = 9°, voxel size = 1 × 1 × 1 mm^3^).

### 2.4 fMRI image preprocessing

Imaging preprocessing and statistical analyses were performed using SPM8 (http://www.fil.ion.ucl.ac.uk/spm/). The first five volumes of each run were discarded to allow for saturation of the signal. Preprocessing consisted of slice‐timing correction, motion correction, coregister to the structural image, normalization of the mean functional image to the MNI template and smoothing with a Gaussian kernel (8 mm FWHM).

### 2.5 Regions of interest and network definition

**FIGURE 2:**
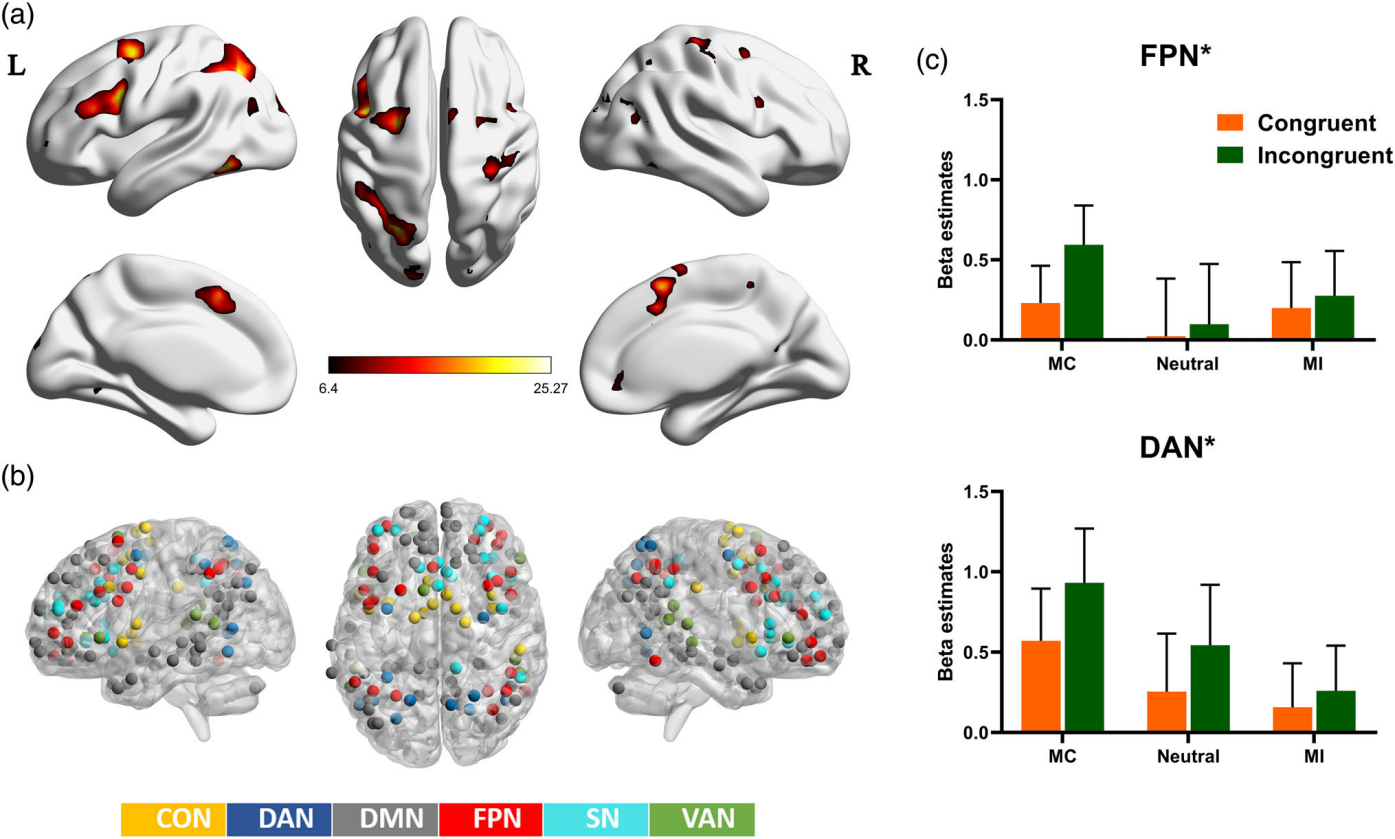
Results of fMRI analysis for the interaction between congruency and context. (a) Brain regions involved in the interaction between congruency and context (*p* < .05, false discovery rate [FDR] corrected). (b) Network nodes included in the network analyses. (c) Mean activations for two of six networks for six task conditions. DAN, dorsal attention network; FPN, fronto‐parietal network. Error bars refer to *SE*. Asterisks indicate significant interaction between the context and congruency. Bonferroni correction was used for multiple comparisons with *p* < .05

The regions of interest (ROIs) and networks in the current study were selected independently of the activity results, thus improving the reliability of the results and avoiding the problem of “double dipping” in data analyses (Kriegeskorte, Simmons, Bellgowan, & Baker, 2009). More specifically, the ROIs and networks were taken from Power atlas (Power et al., 2011), which provides higher test–retest reliability for global and local network properties and has been widely applied in numerous studies. Based on a previous study (Cole et al., 2013) that assigned the original networks of 264 regions of interest to 10 networks of 227 regions of interest based on the Power atlas, we extracted six networks related to the attentional control (Figure 2b): the DMN (58 ROIs), the FPN (25 ROIs), the CON (14 ROIs), the SN (18 ROIs), the VAN (9 ROIs), and the DAN (11 ROIs). All 135 ROIs were defined as a sphere with a radius of 6 mm. Detailed information about the networks and ROIs can be found in Supplementary Table 1.

### 2.6 Statistical analysis

A general‐linear model (GLM) was used to estimate task effects for each participant. The model regressors included temporal onsets for correct responses to each of the six trial types: 3 (context: MC, neutral, MI) × 2 (congruency: congruent, incongruent) analysis. Error trials were modeled as regressors of noninterest. Task regressors were convolved using the canonical hemodynamic response function. In addition, the 24 parameters of head motion, global mean signals within the whole brain, white matter, and cerebrospinal fluid masks, and six run constants were included as covariates in the model. High‐pass filtering (cutoff, 128 s) was used to remove frequency effects.

For each participant, we created contrast images for all regressors of interest. These individual contrast images were then submitted to random‐effects analysis of variance (ANOVA) (flexible factorial design implemented) using context (MC, neutral, MI) and congruence (congruent, incongruent) as factors. The results were submitted to a threshold of *p* < .05 corrected for multiple comparisons (false discovery rate, cluster size >20 voxels).

For network activation analysis, we extracted the beta value for each ROI and condition of interest. Subsequently, the mean values for the six networks were computed by averaging across all ROIs within each network. Finally, the 3 × 2 ANOVA described above was applied to each network and the significance threshold was determined by Bonferroni correction for multiple comparisons with *p* < .05/6.

Furthermore, a PPI analysis was performed, based on the recently developed codes (Di & Biswal, 2019) using SPM8, to assess the brain network interactions associated with the conflict processing under different contexts. First, the time course of the ROI was extracted as the physiological variable and the congruent and incongruent conditions across different contexts were defined as the psychological variables. Together, these variables created the PPI term reflecting regional activity induced by the task. Next, a GLM was generated to estimate the task‐dependent effects of one ROI on another for each region across each of the six conditions (3 [context: MC, neutral, MI] × 2 [congruency: congruent, incongruent]) according to the following formula:

$$Y=\beta_{0}+\beta_{1}X_{1}+\beta_{2}X_{2}+\beta_{3}\left(X_{1}\times X_{2}\right)+e$$

where X_1_ is the time series of a source ROI; X_2_ is the psychological regressor representing a task condition; and (X_1_ × X_2_) is the PPI term between the psychological regressor and the time series of the ROI. This model used the PPI term corresponding to the source region as the explanatory variable (i.e., source, or variable exerting influence) to predict the activity within the target regions as the dependent variable (i.e., target, or region being influenced). This procedure was repeated for each pair of regions and for each task condition, to generate a 135 × 135 symmetric connectivity matrix for each participant and each condition. Specifically, a total of (135 × 134/2 = 9,045) connections with the corresponding estimated parameters (β_3_) were stored in elements of the matrix.

For network level connections, the connectivity change of each intranetwork was computed by averaging the β values of all pairs of regions in each specific network and the internetwork connection was computed by averaging the β values of all ROI pairs pertaining to different networks. The network connectivity data could therefore be analyzed with a 3 (context: MC, neutral, MI) × 2 (congruency: congruent, incongruent) ANOVA to all network pairings and networks. The significance threshold was set at *p* < .05/21 (6 intranetworks and 15 internetworks) using a Bonferroni correction for multiple comparisons in network level.

Finally, we performed correlation analyses between reaction times and connectivity change for all intranetwork and internetwork across different contexts in congruent and incongruent condition separately to further explore how connectivity changes relate to behavioral data.

## 3 RESULTS

### 3.1 Results of the behavioral analysis

A 3 (context: MC, neutral, MI) × 2 (congruency: congruent, incongruent) repeated‐measures ANOVA was performed separately on the reaction time of correct trials and accuracy. The results on RTs showed a main effect of congruency (*F*(1, 29) = 152.59, *p* < .001, *η* ^2^ = 0.84) (Figure 1b and Table 1), as participants responded faster in congruent trials (mean ± *SEM*: 655 ± 15 ms) compared with incongruent trials (745 ± 20 ms). As expected, the interaction between the congruency effects and context was also significant (*F*(2, 58) = 26.81, *p* < .001, *η* ^2^ = 0.48) and was driven by a larger interference effect for the neutral (group mean_*incongruent*_ – group mean_*congruent*_: 80 ms) than for the MI (65 ms) block, and a larger interference effect for the MC (123 ms) than for the neutral (80 ms) block, indicating that the congruence effect was modulated by the context. A main effect of context was not observed (*F*(2, 58) = 0.21, *p* = .81, *η* ^2^ = 0.007). Analyses of accuracy showed that there was no significant main effect of congruency, context, or two‐way interaction (context, *F*(2, 58) = 0.875, *p* = .42, *η* ^2^ = 0.29; congruency, *F*(1, 29) = 3.559, *p* = .069, *η* ^2^ = 0.109; interaction, *F*(2, 58) = 1.73, *p* = .19, *η* ^2^ = 0.056).

**TABLE 1:** Response time (ms) and accuracy (%) for task conditions

	MC		Neutral		MI	
	Congruent trial	Incongruent trial	Congruent trial	Incongruent trial	Congruent trial	Incongruent trial
Accuracy (%)	84 ± 12	87 ± 7	86 ± 10	87 ± 6	86 ± 10	87 ± 7
Response time (ms)	642 ± 87	765 ± 134	657 ± 95	738 ± 110	668 ± 89	733 ± 106

### 3.2 Activation changes

We found that the different context manipulations that affected conflict resolution were supported by the frontal and parietal regions that are involved in cognitive control, including the bilateral dorsolateral prefrontal cortex (dlPFC), bilateral middle frontal gyrus, superior parietal lobule (SPL), posterior parietal cortex (PPC), posterior cingulate cortex, pre‐supplementary motor area, ACC, caudate, and bilateral inferior parietal lobule (Figure 2a and Table 2).

**TABLE 2:** Brain regions associated with interaction between congruency and context

		MNI coordinates					
Region	Hemisphere	*x*	*y*	*z*	No. voxels	Peak *F*	*p*‐Corrected
Dorsolateral prefrontal cortex	L	−45	9	30	1,026	25.27	5.09 × 10^−6^
Middle frontal gyrus	L	−24	0	57		22.21	5.94 × 10^−6^
Supplementary motor area	R	3	15	54		21.51	6.04 × 10^−6^
Inferior temporal gyrus	L	−48	−57	−12	110	24.85	5.09 × 10^−6^
Fusiform gyrus	L	−36	−45	−18		8.26	1.08 × 10^−2^
Superior parietal lobule	L	−27	−57	45	467	23.43	5.38 × 10^−6^
Posterior parietal cortex	L	−24	−69	51		20.55	8.50 × 10^−6^
Inferior parietal lobule	L	−36	−45	39		17.10	4.63 × 10^−5^
Precentral gyrus	R	33	−30	63	189	17.91	3.03 × 10^−5^
Postcentral gyrus	R	57	−12	45		11.01	1.77 × 10^−3^
Postcentral gyrus	R	45	−21	54		10.40	2.65 × 10^−3^
Inferior temporal gyrus	R	54	−54	−15	28	12.31	7.37 × 10^−4^
Lateral occipital gyrus	L	−15	−96	21	79	12.19	7.95 × 10^−4^
Cuneus	L	−15	−87	30		9.50	4.73 × 10^−3^
Dorsolateral prefrontal cortex	R	45	9	27	60	12.02	8.79 × 10^−4^
Middle frontal gyrus	R	54	15	36		9.93	3.57 × 10^−3^
Middle frontal gyrus	R	24	0	57	58	11.13	1.64 × 10^−3^
Middle frontal gyrus	R	39	0	45		6.40	3.56 × 10^−2^
Superior parietal lobule	R	9	−39	54	24	10.81	2.00 × 10^−3^
Middle temporal gyrus	L	−39	−72	21	64	10.31	2.83 × 10^−3^
Middle temporal gyrus	L	−45	−78	21		7.94	1.34 × 10^−2^
Posterior cingulate cortex	R	12	−54	18	20	10.06	3.29 × 10^−3^
Parietal operculum	R	45	−66	18	90	9.82	3.84 × 10^−3^
Middle temporal gyrus	R	42	−72	27		8.53	9.07 × 10^−3^
Superior temporal gyrus	R	51	−60	15		8.26	1.08 × 10^−2^
Anterior cingulate cortex	R	9	39	0	35	9.62	4.36 × 10^−3^
Straight gyrus	R	3	39	−12		7.67	1.61 × 10^−2^
Cuneus	R	21	−87	30	41	9.11	6.13 × 10^−3^
Lateral occipital gyrus	R	18	−93	24		7.64	1.65 × 10^−2^
Posterior parietal cortex	R	12	−75	30		6.70	2.99 × 10^−2^
Middle frontal gyrus	L	−39	57	6	22	8.55	8.97 × 10^−3^
Superior parietal lobule	R	30	−60	51	24	8.41	9.86 × 10^−3^
Inferior parietal lobule	R	33	−51	48		6.76	2.88 × 10^−2^
Fusiform gyrus	L	−30	−60	−9	20	8.01	1.28 × 10^−2^

Abbreviations: L, left hemisphere; MNI, coordinates according to the Montreal Neurological Institute system; R, right hemisphere.

The results of the network activation analysis further showed that the interaction between context and congruency effect was significant within the DAN (*F*(2, 58) = 9.328, *p* < .001, *η* ^2^ = 0.243) and FPN (*F*(2, 58) = 9.115, *p* < .001, *η* ^2^ = 0.239) (Figure 2c), but not within others.

In addition, the ANOVA revealed a main effect of congruency effect within the CON (*F*(1, 29) = 55.179, *p* < .001, *η* ^2^ = 0.655), DAN (*F*(1, 29) = 54.913, *p* < .001, *η* ^2^ = 0.654], DMN (*F*(1, 29) = 57.363, *p* < .001, *η* ^2^ = 0.664), FPN (*F*(1, 29) = 23.902, *p* < .001, *η* ^2^ = 0.452), and SN (*F*(1, 29) = 38.383, *p* < .001, *η* ^2^ = 0.570), but not in VAN (*F*(1, 29) = 0.252, *p* = .619, *η* ^2^ = .009). Specifically, activation of CON, DAN, DMN, and FPN during incongruent trials was consistently larger than it was during congruent trials, except SN of which activation was weaker in incongruent trials than it was in congruent trials. A main effect of context was not found within these six networks.

Using a post hoc analysis, we further examined the interference effect relative to different contexts as well as the context effect relative to specific congruency level. Results indicated significantly stronger activation during incongruent trials than during congruent trials across three contexts for intranetworks of DAN, DMN, and SN. However, context effect on different trial types did not reach a significant level. In particular, the FPN, which is considered as a core network of reactive control, showed a significant congruency effect in the MC context (*F*(1, 29) = 26.964, *p* < .001, *η* ^2^ = 0.482) driven by larger activation during incongruent trials (0.594 ± 0.245) versus congruent trials (0.231 ± 0.232); however, there was a lack of congruency effect in the neutral (*F*(1, 29) = 3.016, *p* = .093, *η* ^2^ = 0.094) or the MI (*F*(1, 29) = 1.914, *p =* .177, *η* ^2^ = 0.062) contexts. This finding is highly consistent with the results of previous studies (Aben et al., 2019).

### 3.3 Connectivity changes

**FIGURE 3:**
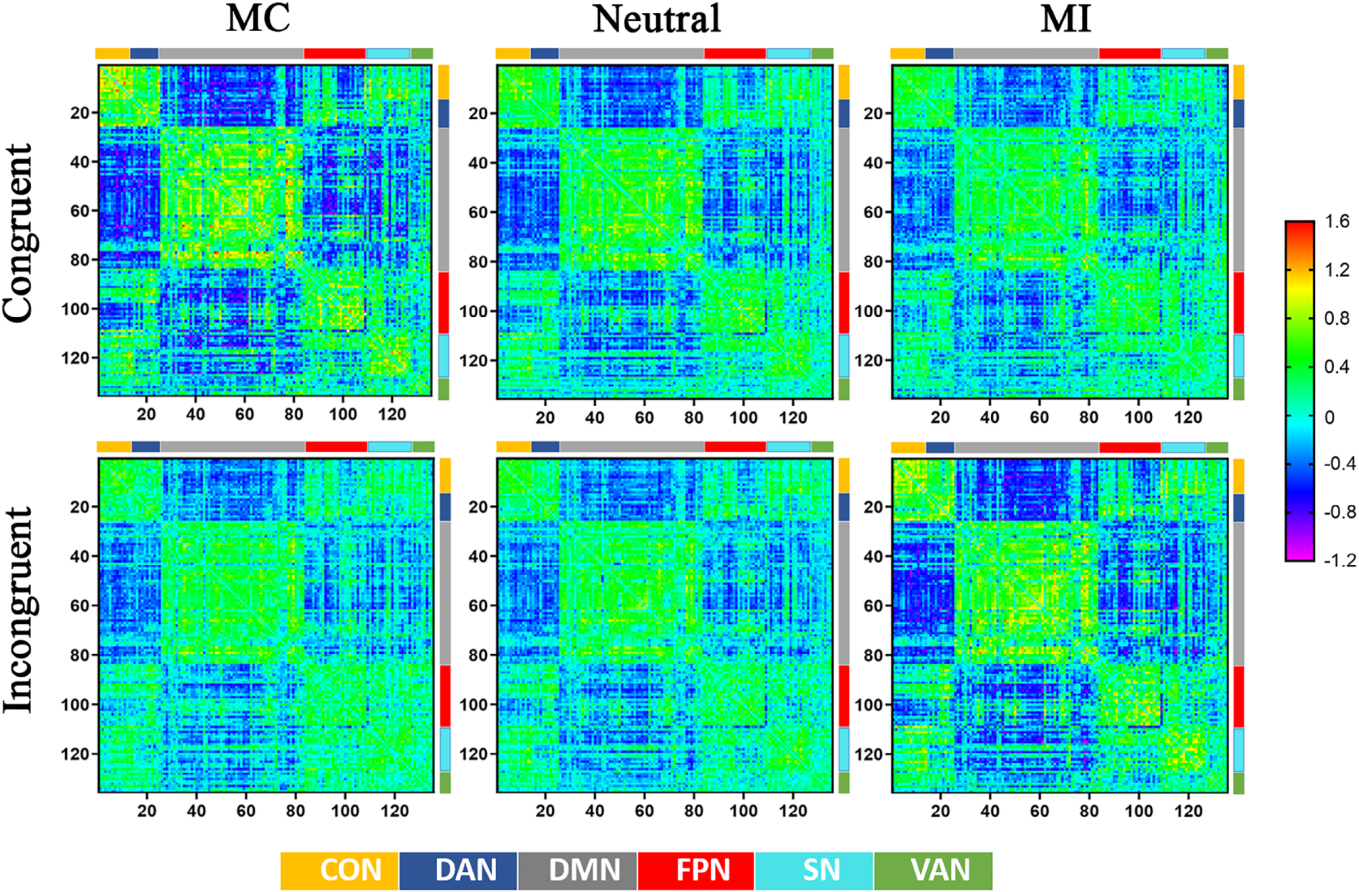
Brain connectivity changes across the six conditions. The colored elements in the matrices indicate the connectivity values, as indicated by the rainbow‐colored bar to the right. The smaller color bars on the top and right sides of each matrix represent the six functional brain networks: (1) cingulo‐opercular, (2) dorsal attention, (3) default mode, (4) fronto‐parietal, (5) salience, and (6) ventral attention

The raw connectivity matrices of PPI effects across the 135 ROIs in the six conditions are displayed in Figure 3. The connectivity pattern of large‐scale networks was modulated by the interaction of context and conflict condition. As the proportion of incongruent trials increased, absolute connectivity strength exhibited an overall trend toward enhancement (i.e., more positive or negative connection) in incongruent trials but attenuation in congruent trials.

**FIGURE 4:**
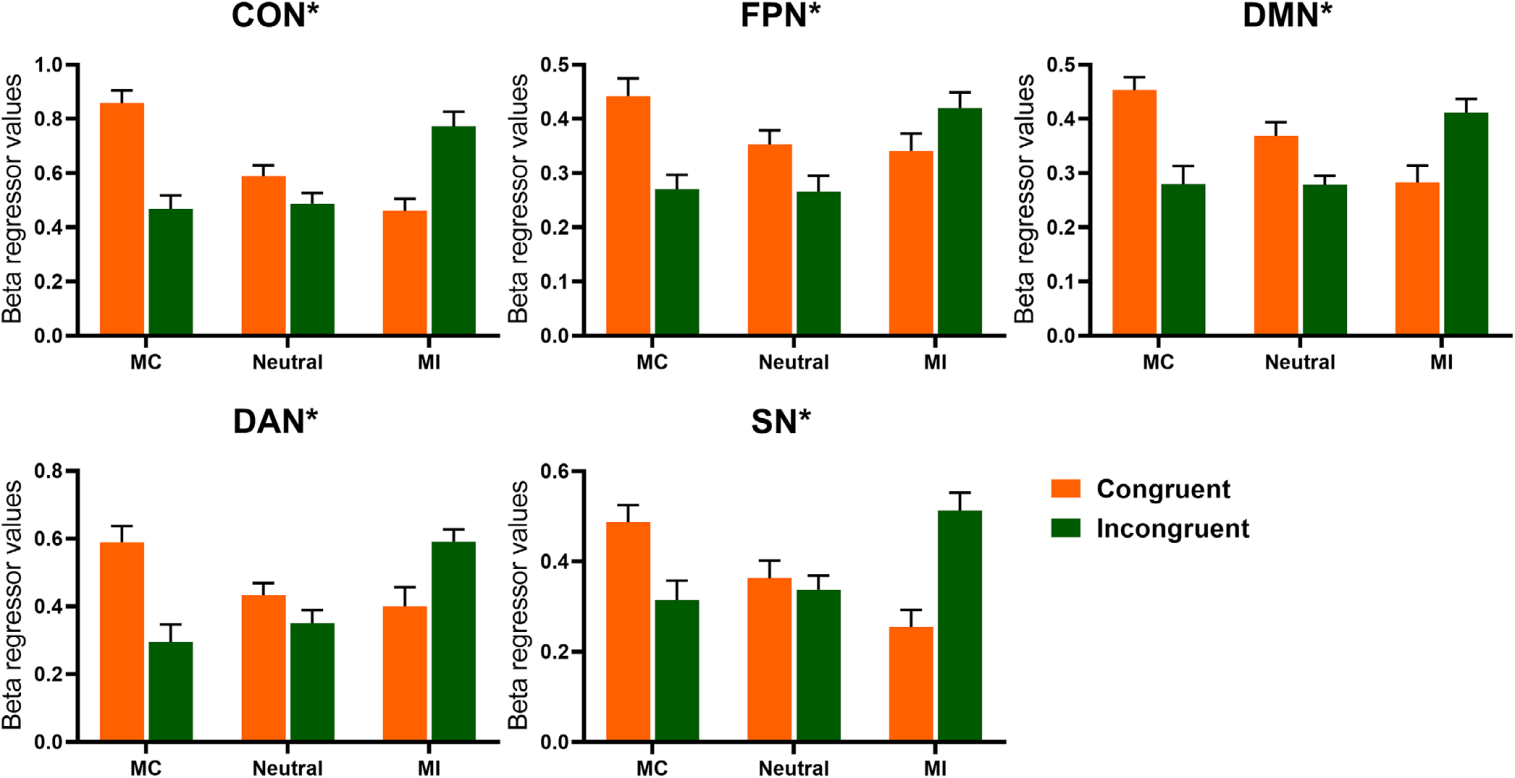
Mean connectivity changes for intranetworks modulated by interaction of context and congruency. Error bars refer to *SE*. Asterisks indicate significant interaction between the context and congruency. Bonferroni correction was used for multiple comparisons with *p* < .05

The within‐network analysis revealed a significant interaction between context and congruency effect for five intranetworks (CON (*F*(2, 28) = 32.573, *p* < .001, *η* ^2^ = 0.529), DAN (*F*(2, 28) = 13.591, *p* < .001, *η* ^2^ = 0.319), DMN (*F*(2, 28) = 18.490, *p* < .001, *η* ^2^ = 0.389), FPN(*F*(2, 28) = 8.360, *p* = .002, *η* ^2^ = 0.224) and SN (*F*(2, 28) = 17.975, *p* < .001, *η* ^2^ = 0.383). Specifically, connectivity within these five networks increased as the proportion of incongruent trials increased in incongruent trials but decreased in congruent trials (Figure 4). Moreover, the main effect of context on connectivity was significant within DAN (*F*(2, 58) = 7.042, *p* = .002, *η* ^2^ = 0.195), as the connection strength in MI context was stronger than it was in MC context (MC: 0.443 ± 0.036, neutral: 0.392 ± 0.024, MI: 0.496 ± 0.035).

**FIGURE 5:**
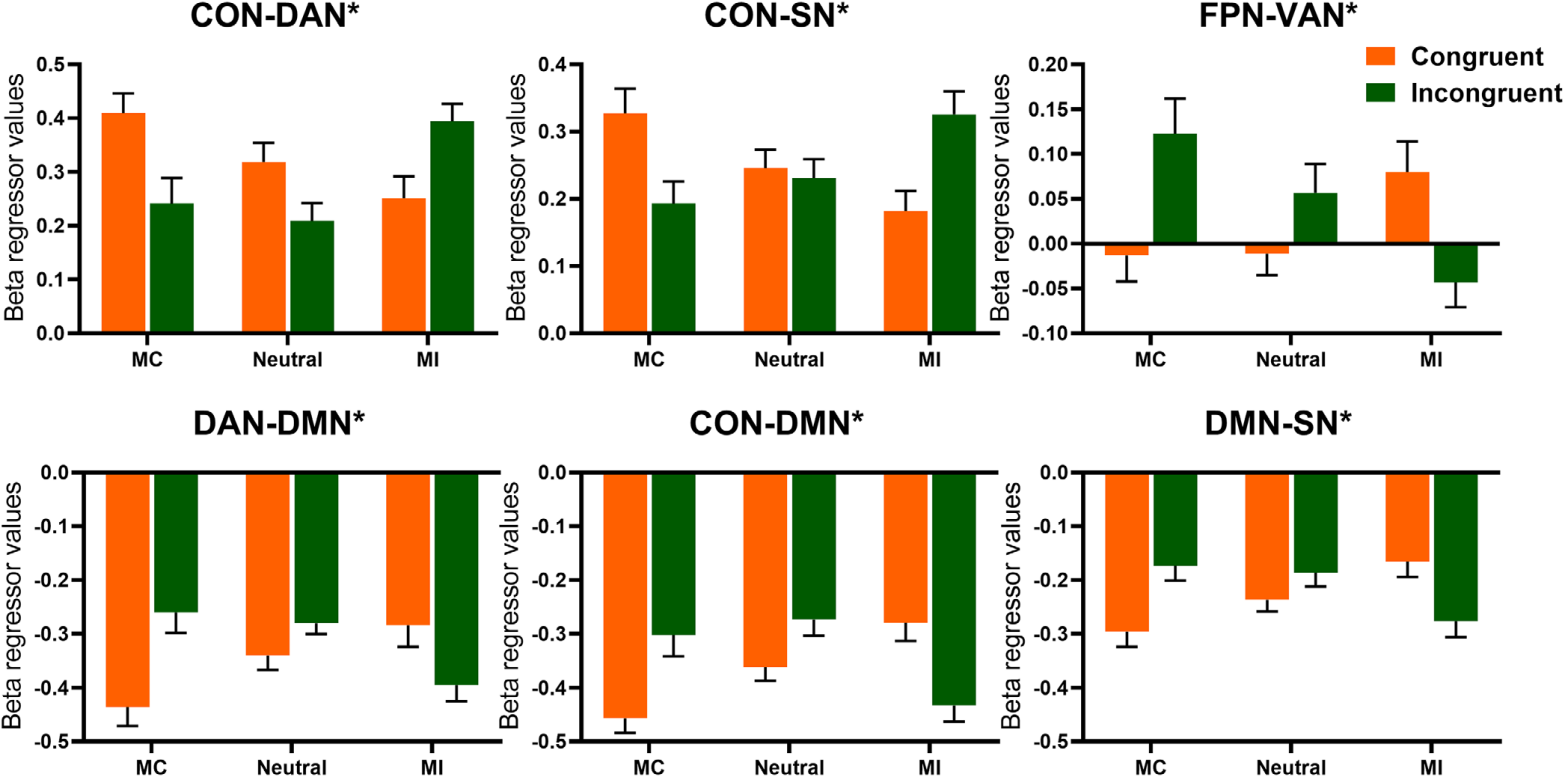
Mean connectivity changes for internetworks modulated by interaction of context and congruency. Error bars refer to *SE*. Asterisks indicate significant interaction between the context and congruency. Bonferroni correction was used for multiple comparisons with *p* < .05

A significant interaction of connectivity between the CON and DAN (*F*(2, 58) = 11.607, *p* < .001, *η* ^2^ = 0.286), between the CON and DMN (*F*(2, 58) = 14.495, *p* < .001, *η* ^2^ = 0.333), between the CON and SN (*F*(2, 58) = 8.850, *p* = .001, *η* ^2^ = 0.234)), between the DAN and DMN (*F*(2, 58) = 9.877, *p* < .001, *η* ^2^ = 0.254), between the DMN and SN (*F*(2, 58) = 8.500, *p* = .001, *η* ^2^ = 0.227), and between the FPN and VAN (*F*(2, 58) = 9.564, *p* < .001, *η* ^2^ = 0.248) was also observed. Specifically, when the proportion of incongruent trials increased, connectivity of the former five internetworks was enhanced in incongruent trials but weakened in congruent trials, whereas the strength of connectivity between the FPN and VAN decreased in incongruent trials but increased in congruent trials (Figure 5). Moreover, neither the main effect of congruency nor the main effect of context on connectivity of any above‐mentioned internetworks reached a significant level after multiple comparison correction.

An additional post hoc analysis for internetwork connectivity between the FPN and VAN revealed that the congruency effect was significant in MC context (*F*(1, 29) = 7.908, *p* = .009, *η* ^2^ = 0.214) and was driven by larger connectivity in incongruent trials (0.123 ± 0.039) versus congruent trials (−0.013 ± 0.029), also significant in MI context (*F*(1, 29) = 9.659, *p =* .004, *η* ^2^ = 0.250) and was driven by stronger connection in congruent trials (0.08 ± 0.034) than in incongruent ones (−0.043 ± 0.028), but not significant in the neutral (*F*(1, 29) = 2.633, *p* = .116, *η* ^2^ = 0.083) context.

### 3.4 Correlation analyses

**FIGURE 6:**
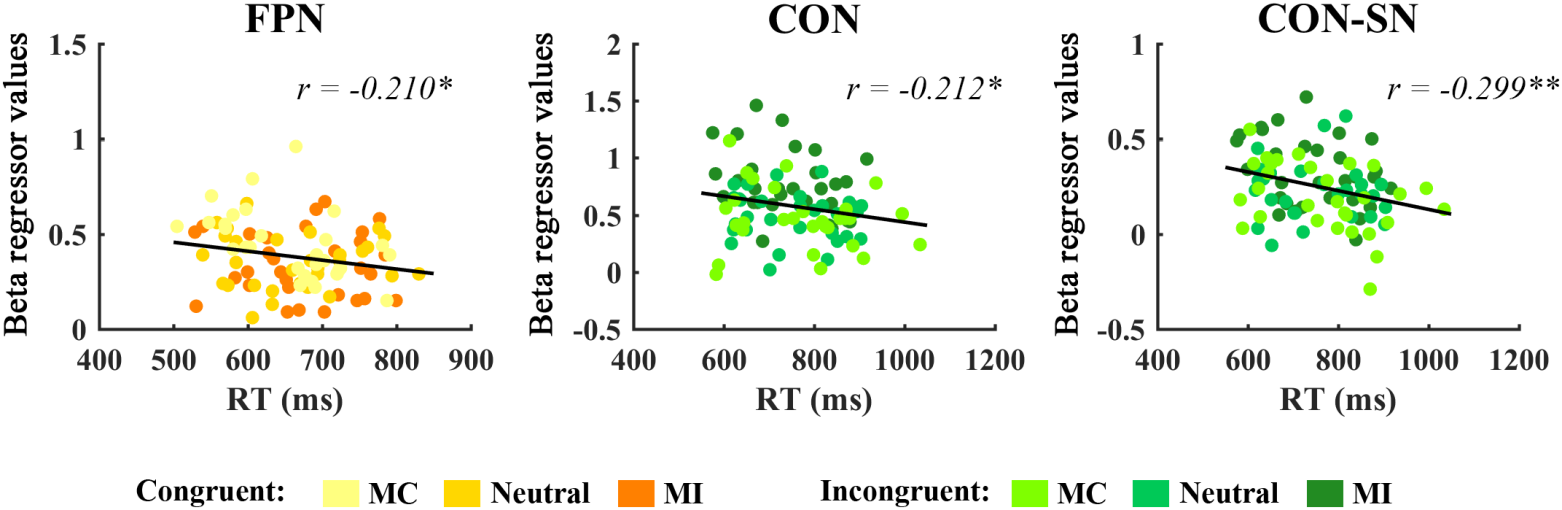
Significant correlations between behavioral reaction times (RT) and intranetwork connectivity change of fronto‐parietal network (FPN) during congruent trials between RT and connectivity change of cingulo‐opercular network (CON) during incongruent trials, and between RT and internetwork connection between the CON and the salience network (SN) during incongruent trials. Asterisks indicate significant correlation. *p < .05, **p < .01, ***p < .001

There were significantly negative correlations between RT and the connectivity for FPN in congruent condition (*r* = −.210, *p* = .047), and for CON in incongruent condition (*r* = −.212, *p* = .045) as well as for internetwork between CON and SN in incongruent condition (*r* = −.299, *p* = .004) (Figure 6).

## 4 DISCUSSION

The present study aimed to examine how the interactions within and between attentional networks serve control demand, which was manipulated by the proportion of congruency in block level. Human behavior showed a robust interference effect, which was characterized by a longer RT of incongruent trials relative to congruent ones. Importantly, the interference effect was affected by the proportion of incongruence, as declining interference effects were found as the frequency of incongruent trials increased in blocks, indicating a contextual effect on cognitive control. In line with previous reports, the fMRI study performed here indicated that the activation of frontal and parietal regions, such as the bilateral dlPFC, PPC, ACC, and SPL, contributes to the interaction between congruency and context (Aben et al., 2019; Grandjean et al., 2013; Xia, Li, & Wang, 2016). A follow‐up analysis showed that activation of the DAN and FPN exhibited a larger response to the incongruent trials than to the congruent trials. Moreover, as the proportion of incongruent trials increased within a block, functional connectivity was enhanced within the CON, FPN, DMN, DAN, and SN and was accompanied by strengthened coupling between the CON and DAN/SN and between the DMN and CON/DAN/SN, but weakened coupling between the FPN and VAN. We further found that connectivity reinforcement within the CON, within the FPN, and between the CON and SN were positively associated with improvement in behavioral performance.

Active internal representations of experience and contextual information in working memory play a critical role in guiding goal‐directed behavior. The participants used the prediction of control demand (e.g., anticipated conflict or congruency levels on upcoming trials), which is derived from previous experience of conflict and contextual information to drive strategic upregulation in top‐down control, via the biasing of information processing to favor the task‐relevant dimension and to deviate from the task‐irrelevant dimension (Chiu & Egner, 2019; Jiang et al., 2014; Jiang et al., 2015). In the MI context, participants were able to use frequencies to predict that the incongruent trials are most likely to occur next and then enhanced the top‐down biasing of the task‐specific dimension over the task‐irrelevant dimension. In contrast, in the MC context, the word is strongly predictive of the correct response. In addition, our results are in line with previous studies reporting that a variation of the conflict effects under the manipulation of the context in the activity of frontal and parietal regions (Aben et al., 2019; Grandjean et al., 2013), indicating that the activity of frontal and parietal relevant brain networks is associated with top‐down biasing of information processing. Notably, the activation analysis confirmed that there were significant interactions between context and the congruency effect for two large‐scale brain networks: the DAN, and FPN. These results are consistent with the view that the frontal and parietal brain networks play a vital role in cognitive control.

In addition, the enhanced top‐down biasing of task‐relevant processing was indexed by increasing functional connectivity within the CON, DAN, DMN, FPN, and SN. Moreover, the CON connected more strongly with the DAN, SN, and DMN, whereas the FPN connected more strongly with the VAN. The CON and DAN are believed to underpin the stable maintenance of the tasks that are set throughout long‐term trials (Cocchi et al., 2013). Thus, the enhanced connectivity between the CON and DAN may support the prediction of conflict level and cognitive control, which are frequently needed and should be sustained as the proportion of incongruent trials increases within a block. In fact, previous studies demonstrated that well‐practiced S‐R associations are accompanied by enhanced coupling between the CON and DAN (Mohr et al., 2016; Mohr, Wolfensteller, & Ruge, 2018). In addition, as control demand increased, the enhanced connectivity observed between the CON and SN may indicate that cognitive processing in the CON is closely correlated with stable saliency of the stimulus feature. Moreover, efficient facilitation of task‐relevant external processing in the control networks and suppression of task‐irrelevant internal processing in the DMN as a function of increased task demands are thought to be critical for optimal cognitive performance (Elton & Gao, 2015; Hellyer et al., 2014; Kelly, Uddin, Biswal, Castellanos, & Milham, 2008; Leech, Kamourieh, Beckmann, & Sharp, 2011; Spreng, Stevens, Chamberlain, Gilmore, & Schacter, 2010). In the current study, we also found that the coupling between the DMN and CON/DAN/SN showed a more negative response as the proportion of incongruent trials increased. In contrast with that observed for the CON and DAN, the FPN and VAN play a crucial role in orienting attention to task‐relevant perceptual input and trial‐specific adaptive control (Cocchi et al., 2013; Gratton et al., 2017; Kim, 2014; Long & Kuhl, 2018). As conflict frequency decreased from MI to MC, the enhanced connectivity between the FPN and VAN contributed to rapid conflict resolution in the Stroop task.

Furthermore, the dual mechanisms of control (DMC) framework predict that cognitive control can work on different time scales (Braver, 2012). Proactive control leads to the preparatory biasing of attention based on advance information (e.g., anticipated conflict or congruency levels on upcoming trials) (Braver, 2012; Braver, Reynolds, & Donaldson, 2003). This biasing may derive from the active maintenance of context representations and task goals and is thought to be associated with sustained activity within the CON (Dosenbach et al., 2006; Dosenbach et al., 2007; Palenciano et al., 2019). In contrast, reactive control implements a transient control that acts in response to changing environmental demands based on the retrieval of goal‐relevant information (Braver, 2012; Braver et al., 2003). This reactive control mechanism is thought to be accompanied by the activity of the FPN (Dosenbach et al., 2006; Dosenbach et al., 2007; Palenciano et al., 2019). These two mechanisms cooperate to underlie cognitive control. In this study, on the one hand, sustaining control was necessary to support the prediction of conflict level to achieve optimal task performance in MI blocks, as stronger connectivity within the CON and coupling between the CON and DAN/SN/DMN were observed in the MI context versus the MC context. On the other hand, reactive control was thought to be more active when incongruent trials were rare in the MC context. The current results agree with this dual‐network perspective in the sense that the FPN showed significantly larger activation and the internetwork coupling between the FPN and VAN showed significantly larger strength for incongruent trials in the MC context than in the MI context. To summarize, our results are largely consistent with this framework for the neural mechanism underlying the flexible cognitive control.

One major limitation of this study might be that healthy participants were recruited by their oral report of no psychiatric or neurological disorders, rather than the use of screening instruments. Previous studies suggested the various degree of impairment of conflict adaptation in patients (Abrahamse et al., 2016). For example, the deficits in conflict adaptation in individuals at familial risk for developing bipolar disorder (Patino et al., 2013), and the reduction in the use of proactive control in individuals with schizophrenia (Braver, 2012). Nonetheless, there were several findings showing the significant behavioral PC effect in Parkinson's disease (Ruitenberg, Abrahamse, Santens, & Notebaert, 2019) and schizophrenia (Henik et al., 2002). In the present study, the PC effect was observed in both behavioral and fMRI data, but the potential influences from personality traits or mental states cannot be ruled out. It is significant for future studies to explore the potential relationship between the PC effect and clinical and developmental populations or groups.

In summary, the current study demonstrated that participants are better at selecting appropriate cognitive or motor actions based on the prediction of the control demand, which is derived from previous experience of conflict and contextual information. This ability is supported by functional connectivity within and between the attentional networks that are used to make control adjustments as needed across entire task periods. These findings advance our understanding of the interactions of attentional and control networks in the flexible regulation of cognitive control.

## CONFLICT OF INTERESTS

The authors declare no potential conflict of interest.

## Supporting information


**Appendix** **S1**: Supporting information

## Data Availability

Data supporting the findings of this study are available on request from the corresponding author on reasonable request.
