# Supplementary material for: Large‐scale reconfiguration of connectivity patterns among attentional networks during context‐dependent adjustment of cognitive control
Source: Hum Brain Mapp. 2021 May 14;42(12):3821–32. doi: 10.1002/hbm.25467 (PMC8288082; doi:10.1002/hbm.25467)
Supplement: Supplementary file 1 — Appendix S1: Supporting information [file HBM-42-3821-s001.docx]

**Supplementary Information**

**Supplementary Table 1.** Network nodes included in the network analyses. Coordinates and network assignments were taken from Power et al.^1^ and Cole et al.^2^.

| Network name | Network-No. |
| --- | --- |
| Default Mode Network | 1 |
| Fronto-Parietal Network | 2 |
| Cingulo-Opercular Network | 6 |
| Salience Network | 7 |
| Ventral Attention Network | 8 |
| Dorsal Attention Network | 9 |

| Node-No. | MNI x | MNI y | MNI z | Network-No. |
| --- | --- | --- | --- | --- |
| 1 | -3 | 3 | 54 | 6 |
| 2 | 54 | -27 | 33 | 6 |
| 3 | 18 | -9 | 63 | 6 |
| 4 | -15 | -6 | 72 | 6 |
| 5 | -9 | -3 | 42 | 6 |
| 6 | 36 | 0 | -3 | 6 |
| 7 | 12 | 0 | 69 | 6 |
| 8 | 6 | 9 | 51 | 6 |
| 9 | -45 | 0 | 9 | 6 |
| 10 | 48 | 9 | 0 | 6 |
| 11 | -33 | 3 | 3 | 6 |
| 12 | -51 | 9 | -3 | 6 |
| 13 | -6 | 18 | 33 | 6 |
| 14 | 36 | 9 | 0 | 6 |
| 15 | -42 | -75 | 27 | 1 |
| 16 | 6 | 66 | -3 | 1 |
| 17 | 9 | 48 | -15 | 1 |
| 18 | -12 | -39 | 0 | 1 |
| 19 | -18 | 63 | -9 | 1 |
| 20 | -45 | -60 | 21 | 1 |
| 21 | 42 | -72 | 27 | 1 |
| 22 | -45 | 12 | -33 | 1 |
| 23 | 45 | 15 | -30 | 1 |
| 24 | -69 | -24 | -15 | 1 |
| 25 | -45 | -66 | 36 | 1 |
| 26 | -39 | -75 | 45 | 1 |
| 27 | -6 | -54 | 27 | 1 |
| 28 | 6 | -60 | 36 | 1 |
| 29 | -12 | -57 | 15 | 1 |
| 30 | -3 | -48 | 12 | 1 |
| 31 | 9 | -48 | 30 | 1 |
| 32 | 15 | -63 | 27 | 1 |
| 33 | -3 | -36 | 45 | 1 |
| 34 | 12 | -54 | 18 | 1 |
| 35 | 51 | -60 | 36 | 1 |
| 36 | 24 | 33 | 48 | 1 |
| 37 | -9 | 39 | 51 | 1 |
| 38 | -15 | 30 | 54 | 1 |
| 39 | -36 | 21 | 51 | 1 |
| 40 | 21 | 39 | 39 | 1 |
| 41 | 12 | 54 | 39 | 1 |
| 42 | -9 | 54 | 39 | 1 |
| 43 | -21 | 45 | 39 | 1 |
| 44 | 6 | 54 | 15 | 1 |
| 45 | 6 | 63 | 21 | 1 |
| 46 | -6 | 51 | 0 | 1 |
| 47 | 9 | 54 | 3 | 1 |
| 48 | -3 | 45 | -9 | 1 |
| 49 | 9 | 42 | -6 | 1 |
| 50 | -12 | 45 | 9 | 1 |
| 51 | -3 | 39 | 36 | 1 |
| 52 | -3 | 42 | 15 | 1 |
| 53 | -21 | 63 | 18 | 1 |
| 54 | -9 | 48 | 24 | 1 |
| 55 | 66 | -12 | -18 | 1 |
| 56 | -57 | -12 | -9 | 1 |
| 57 | -57 | -30 | -3 | 1 |
| 58 | 66 | -30 | -9 | 1 |
| 59 | -69 | -42 | -6 | 1 |
| 60 | 12 | 30 | 60 | 1 |
| 61 | 12 | 36 | 21 | 1 |
| 62 | 51 | -3 | -15 | 1 |
| 63 | -27 | -39 | -9 | 1 |
| 64 | 27 | -36 | -12 | 1 |
| 65 | -33 | -39 | -15 | 1 |
| 66 | 27 | -78 | -33 | 1 |
| 67 | 51 | 6 | -30 | 1 |
| 68 | -54 | 3 | -27 | 1 |
| 69 | 48 | -51 | 30 | 1 |
| 70 | -48 | -42 | 0 | 1 |
| 71 | -45 | 30 | -12 | 1 |
| 72 | -9 | 12 | 66 | 8 |
| 73 | 48 | 36 | -12 | 1 |
| 74 | -45 | 3 | 45 | 2 |
| 75 | 48 | 24 | 27 | 2 |
| 76 | -48 | 12 | 24 | 2 |
| 77 | -54 | -48 | 42 | 2 |
| 78 | -24 | 12 | 63 | 2 |
| 79 | 57 | -54 | -15 | 2 |
| 80 | 24 | 45 | -15 | 2 |
| 81 | 33 | 54 | -12 | 2 |
| 82 | 48 | 9 | 33 | 2 |
| 83 | -42 | 6 | 33 | 2 |
| 84 | -42 | 39 | 21 | 2 |
| 85 | 39 | 42 | 15 | 2 |
| 86 | 48 | -42 | 45 | 2 |
| 87 | -27 | -57 | 48 | 2 |
| 88 | 45 | -54 | 48 | 2 |
| 89 | 33 | 15 | 57 | 2 |
| 90 | 36 | -66 | 39 | 2 |
| 91 | -42 | -54 | 45 | 2 |
| 92 | 39 | 18 | 39 | 2 |
| 93 | -33 | 54 | 3 | 2 |
| 94 | -42 | 45 | -3 | 2 |
| 95 | 33 | -54 | 45 | 2 |
| 96 | 42 | 48 | -3 | 2 |
| 97 | -42 | 24 | 30 | 2 |
| 98 | -3 | 27 | 45 | 2 |
| 99 | 12 | -39 | 51 | 7 |
| 100 | 54 | -45 | 36 | 7 |
| 101 | 42 | 0 | 48 | 7 |
| 102 | 30 | 33 | 27 | 7 |
| 103 | 48 | 21 | 9 | 7 |
| 104 | -36 | 21 | 0 | 7 |
| 105 | 36 | 21 | 3 | 7 |
| 106 | 36 | 33 | -3 | 7 |
| 107 | 33 | 15 | -9 | 7 |
| 108 | -12 | 27 | 24 | 7 |
| 109 | 0 | 15 | 45 | 7 |
| 110 | -27 | 51 | 21 | 7 |
| 111 | 0 | 30 | 27 | 7 |
| 112 | 6 | 24 | 36 | 7 |
| 113 | 9 | 21 | 27 | 7 |
| 114 | 30 | 57 | 15 | 7 |
| 115 | 27 | 51 | 27 | 7 |
| 116 | -39 | 51 | 18 | 7 |
| 117 | 54 | -42 | 21 | 8 |
| 118 | -57 | -51 | 9 | 8 |
| 119 | -54 | -39 | 15 | 8 |
| 120 | 51 | -33 | 9 | 8 |
| 121 | 51 | -30 | -3 | 8 |
| 122 | 57 | -45 | 12 | 8 |
| 123 | 54 | 33 | 0 | 8 |
| 124 | -48 | 24 | 0 | 8 |
| 125 | 9 | -63 | 60 | 9 |
| 126 | -51 | -63 | 6 | 9 |
| 127 | 21 | -66 | 48 | 9 |
| 128 | 45 | -60 | 3 | 9 |
| 129 | 24 | -57 | 60 | 9 |
| 130 | -33 | -45 | 48 | 9 |
| 131 | -27 | -72 | 36 | 9 |
| 132 | -33 | 0 | 54 | 9 |
| 133 | -42 | -60 | -9 | 9 |
| 134 | -18 | -60 | 63 | 9 |
| 135 | 30 | -6 | 54 | 9 |

1. Power, J. D. *et al.* Functional Network Organization of the Human Brain. *Neuron* **72,** 665–678 (2011).

2. Cole, M. W. *et al.* Multi-task connectivity reveals flexible hubs for adaptive task control. *Nat. Neurosci.* **16,** 1348–1355 (2013).
